# IgG4-related kidney disease – an update

**DOI:** 10.1097/MNH.0000000000000102

**Published:** 2015-02-11

**Authors:** Mitsuhiro Kawano, Takako Saeki

**Affiliations:** a Division of Rheumatology, Kanazawa University Hospital, Kanazawa, Japan; b Department of Internal Medicine, Nagaoka Red Cross Hospital, Nagaoka, Japan

**Keywords:** IgG4, IgG4-related disease, membranous glomerulonephritis, tubulointerstitial nephritis

## Abstract

**Purpose of review:**

IgG4-related disease (IgG4-RD) is a recently recognized systemic inflammatory disorder that can affect most organs/tissues such as sarcoidosis. The kidney is a frequently affected organ with tubulointerstitial nephritis (TIN), the representative lesion of IgG4-RD. This review focuses on the latest knowledge of IgG4-related kidney disease (IgG4-RKD).

**Recent findings:**

A wide range of renal manifestations of IgG4-RD, that is TIN, membranous glomerulonephritis (MGN) and other glomerular lesions, and pyelitis, are collectively referred to as IgG4-RKD. Clinically, decreased renal function, or characteristic imaging findings such as multiple low-density lesions on contrast-enhanced computed tomography or diffuse thickening of the renal pelvic wall, are typical presenting features. Although a rapid response to corticosteroid therapy is a very important feature of IgG4-TIN, in cases in which renal function is moderately to severely decreased before therapy, only partial recovery of renal function is obtained.

**Summary:**

TIN with characteristic imaging findings is a typical manifestation of IgG4-RKD in the interstitium, while MGN is a representative manifestation of the glomerular lesions. Although IgG4 is a central feature of IgG4-RD, the recent discovery of IgG4-negative IgG4-RD raises questions about the causative role of the IgG4 molecule in this context.

## INTRODUCTION

IgG4-related disease (IgG4-RD), a recently recognized systemic inflammatory disorder, generally presents as a mass-forming lesion, or lesions, or organ enlargement [1,2]. Clinical symptoms are diverse depending on the combination of organs affected, but most patients have only mild or no symptoms. The most important feature is marked IgG4-positive plasma cell (IgG4+PC) infiltration in affected organs [3,4]. In addition, it has common histopathological features: dense lymphoplasmacytic infiltrates, storiform fibrosis and obliterative phlebitis [4]. Lacrimal and salivary glands, pancreas, kidneys and aorta/retroperitoneum are frequently affected organs. Patients often have multiple organ involvement simultaneously and sometimes metachronously, and spontaneous regression has been observed. Target organs are summarized in Table 1[5–14,15^▪^].

The kidney is a representative organ, and the various renal lesions are collectively referred to as IgG4-related kidney disease (IgG4-RKD) [16–18]. Tubulointerstitial nephritis (TIN) is a typical lesion of the renal parenchyma, named IgG4-related TIN (IgG4-TIN), and renal pyelitis is typical of the renal pelvis [5]. IgG4-related retroperitoneal fibrosis can also induce renal insufficiency through hydronephrosis.  

**Box 1:**
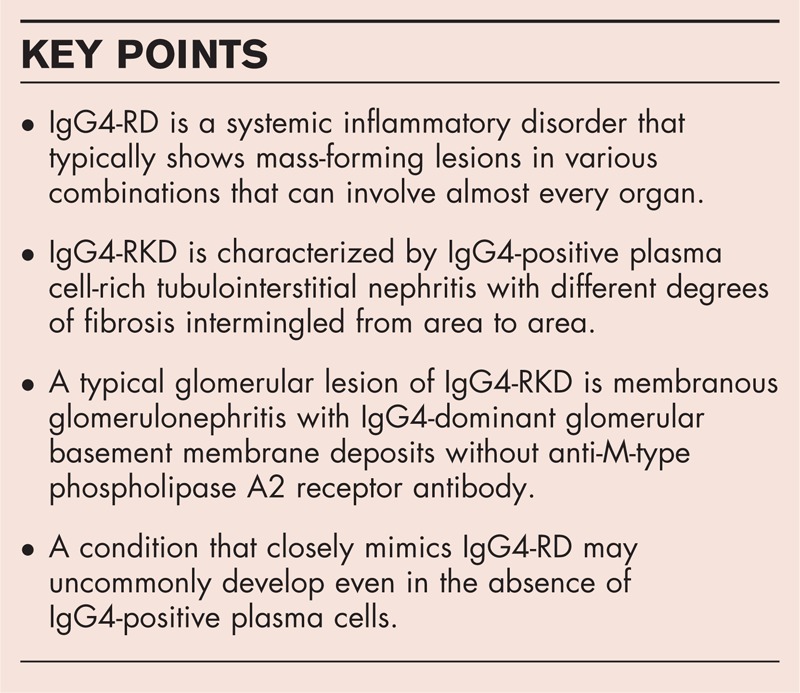
no caption available

## CLINICAL AND LABORATORY FEATURES OF IgG4-RELATED KIDNEY DISEASE

Patients with IgG4-RKD present at an average age of 65 years, and 73–87% are men [16–18]. Two major clinical presentations are well recognized: unexplained renal dysfunction and imaging abnormality. In one study, about half of all patients were suspected of having IgG4-RKD because of renal dysfunction, with renal lesions detected in the remaining patients during the course of imaging evaluation for IgG4-RD [18]. In another study, 77% of patients presented with acute or progressive renal failure requiring renal biopsy [17]. In both studies, more than 80% of patients had other organ involvement.

Elevated serum IgG level and hypocomplementaemia are characteristic features of IgG4-RKD. Although hypocomplementaemia is a distinct feature of IgG4-RD, a relatively low proportion of patients actually have it. Muraki *et al.*[19] evaluated serum complement levels in 44 patients with autoimmune pancreatitis (AIP) and found that only 17% of them had a CH50 of less than 30 U/ml. In contrast, it seems to be more frequent if the kidney is involved, with more than 50% of patients having hypocomplementaemia [17,18]. In cases with IgG4-RKD with hypocomplementaemia, both C3 and C4 are extremely low, resembling the active stage of systemic lupus erythematosus (SLE). In addition, complement might become a biomarker to monitor the recurrence of IgG4-TIN [20^▪▪^,^21^]. Saeki *et al.*[20^▪▪^] followed serum complement levels in 14 patients with IgG4-RKD and found that three again showed a decrease at the time of relapse. Hyper IgG-aemia is also prominent in many cases, and serum IgG levels exceeded 3000 mg/dl in 50% of patients in our IgG4-RKD series [18]. Increased serum levels of IgE and eosinophilia are other features possibly related to the allergic predisposition of this disease [16–18].

Elevated serum IgG4 levels are the most important serological finding in IgG4-RKD [1–3]. Although about 20–30% of patients with IgG4-RD have normal serum IgG4 levels, in two studies more than 90% of patients with IgG4-RKD had increased serum IgG4 levels [17,18]. Serum levels of IgG4 dramatically decrease after successful corticosteroid therapy, but show re-elevation without apparent relapse in about half of the patients during maintenance steroid therapy [20^▪▪^].

Although neither proteinuria nor haematuria is usually detected in IgG4-TIN, many patients with coexistent glomerular lesions have proteinuria or haematuria, or both, and patients with membranous glomerulonephritis (MGN) may show even nephrotic levels of proteinuria. In contrast to drug-induced acute TIN, IgG4-TIN is usually not accompanied by urinary excretion of many white blood cells (WBCs) or WBC casts. This finding probably mirrors a very mild tubulitis, a histopathological feature of IgG4-TIN.

Serum C-reactive protein levels are usually normal, with this being a useful marker to distinguish IgG4-RD from anti-neutrophil cytoplasmic antibody (ANCA)-associated vasculitis or Castleman's disease.

## IMAGING FEATURES OF IgG4-RELATED KIDNEY DISEASE

**FIGURE 1:**
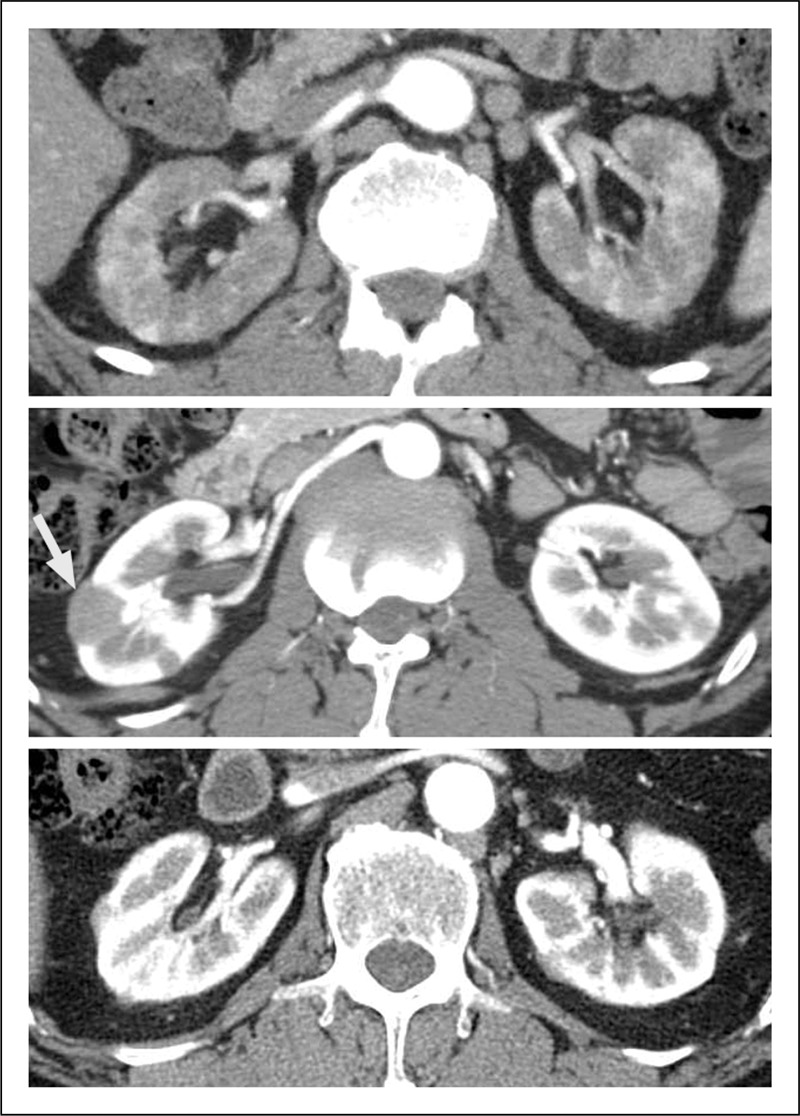
A variety of patterns of multiple low-density lesions on contrast-enhanced computed tomography (CT). Upper: Contrast-enhanced CT scan shows bilateral diffuse patchy involvement. Middle: Contrast-enhanced CT scan shows multiple parenchymal low-density lesions including mass-like lesions protruding beyond the surface of the kidney (arrow). Lower: Contrast-enhanced CT scan shows a rim-like lesion of the kidney.

A distinguishing feature of IgG4-TIN is its characteristic imaging findings frequently observed on computed tomography (CT) [18,22,23]. Contrast-enhanced CT is most useful in delineating the characteristics and distribution of the renal lesions. Multiple or solitary, round or wedge-shaped, parenchymal low-density lesions are common on CT [22,23]. Generally, solitary lesions are very rare, but if encountered, the suspicion of malignant tumour is high and often leads to nephrectomy [24]. In some cases, the lesions are well defined on contrast-enhanced CT, but can be ill defined in others, and in the latter, ‘diffuse patchy involvement’ is a more suitable description in extreme cases (Fig. 1). In addition, mass-like lesions protruding beyond the surface of the kidney, suggestive of tumour progression, may be detected in some cases (Fig. 1). Corresponding to the rim-like lesion of type 1 AIP, a rim-like lesion of the kidney is occasionally seen along a part of the renal capsule (Fig. 1) [22,23]. In addition to parenchymal lesions, renal pelvic lesions are sometimes encountered as a diffuse thickening of the renal pelvis wall with smooth intraluminal surface during systemic evaluation of IgG4-RD by CT [18,22,23].

Recently, MRI has become a useful imaging method to detect IgG4-RKD from a very early stage [23,25]. A typical finding of such lesions is hypointensity on T2-weighted images. Moreover, using diffusion-weighted imaging, a study showed that sensitivity was 100% in an analysis of 20 patients with presumptive IgG4-RKD (14 with contrast enhancement; six without) [25]. Therefore, if impaired renal function contraindicates the use of contrast-enhanced CT, MRI might be a promising alternative.

FDG-PET [26,27] and gallium scintigraphy [28,29] are other imaging modalities sometimes employed. They are used mainly for whole body screening to determine the extent of the systemic organ involvement by IgG4-RD.

## HISTOPATHOLOGICAL FEATURES OF IgG4-RELATED KIDNEY DISEASE: TUBULOINTERSTITIAL LESIONS

**FIGURE 2:**
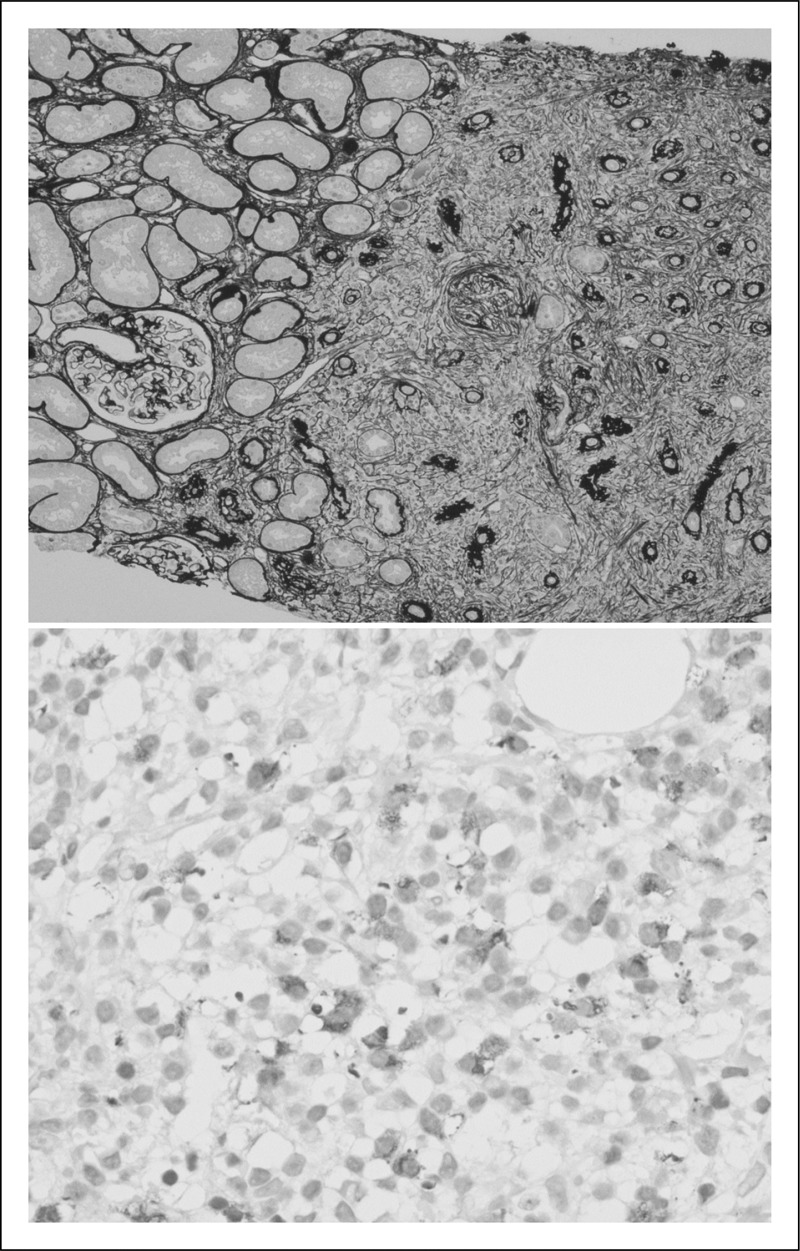
Typical histological features of IgG4-related tubulointerstitial nephritis. Upper: Histopathological examination in a patient with IgG4-related kidney disease (IgG4-RKD) shows plasma cell rich tubulointerstitial nephritis with different stages of fibrosis intermingled within different areas (periodic acid-methenamine-silver staining x100). Lower: Immunostaining for IgG4 shows many IgG4-positive plasma cells in the area of inflammation just outside the renal capsule, probably corresponding to the rim-like lesion of the kidney noted on imaging study (x400).

Plasma cell rich TIN with fibrosis and sometimes numerous infiltrating eosinophils are typical pathological findings of IgG4-RKD (Fig. 2) [4,30–32]. Histologic findings are mandatory for the definite diagnosis of IgG4-RKD. However, several situations such as inaccessible regional lesion distribution (e.g. lesions distributed in only the upper pole of the kidney) hamper histologic examination of the kidney. In such cases, histologic findings from other organs could support typical renal imaging findings and clinical features of IgG4-RKD to allow the diagnosis of IgG4-RKD. Although Sjögren's syndrome sometimes shows plasma cell rich TIN, IgG4 immunostaining clearly differentiates these two diseases. Usually, more than 10 infiltrating IgG4 and plasma cells per high-power field or at least 40% of the ratio of IgG4 and plasma cells to IgG and plasma cells are employed as the cutoff values. However, the specificity of IgG4 immunostaining is not high because ANCA-associated vasculitis, particularly eosinophilic granulomatosis with polyangiitis [33–35] and granulomatosis with polyangiitis (GPA) [36], sometimes show lymphoplasmacytic infiltrates with copious IgG4 and plasma cells in the interstitium. Moreover, serum IgG4 levels have been reported to be sometimes elevated in such cases. Therefore, special caution is needed to differentiate IgG4-RD from ANCA-associated vasculitis. Elevated serum CRP levels and a partial response to corticosteroid therapy seem to be helpful in differentiating these diseases.

Similarly, Houghton and Troxell [37] reported that an abundance of IgG4 and plasma cells in the renal interstitium is not specific for IgG4-TIN but found in some patients with necrotizing glomerulonephritis, diabetic nephropathy, lupus nephritis, MGN and idiopathic TIN. Therefore, it must be kept in mind that IgG4 and plasma cell infiltration is a necessary, but not specific, finding for the diagnosis of IgG4-TIN.

Another important pathological feature is fibrosis [4,16–18,31,32]. At least some portion of the fibrosis shows a storiform pattern, namely a swirling fibrosis. Tubular atrophy with thickened tubular basement membrane (TBM) and disappearance of tubules are prominent in the lesions. In IgG4-TIN, fibrosis is generally more severe than that in other types of TIN, with the degree of fibrosis differing from area to area. Several studies have tried to define the stages of the fibrosis according to its degree, and one study showed that different stages of fibrosis are intermingled within different areas of the needle-biopsied specimens of the same case [38].

The specific distribution of renal parenchyma lesions is another distinctive feature of this disease. First, the margin between affected and unaffected portions is very clearly demarcated, with this finding thought to correspond to the imaging feature of multiple low-density lesions [16,18,32]. Second, lymphoplasmacytic cell infiltration into and beyond the renal capsule is a unique feature of this disease, reflected by the rim-like lesion of the kidney noted on CT (Fig. 2) [32,38].

Granuloma formation and fibrinoid necrosis of the artery have never been reported in IgG4-RKD [17,18], although only one case of IgG4-related renal arteritis without fibrinoid necrosis has been described [39]. Neutrophil infiltration is also very rare, so that the presence of these lesions is a useful clue to rule out IgG4-RKD [18].

Immunoglobulin and complement deposition in the TBM and interstitium has been documented by immunofluorescence microscopy [17,30–32]. Raissian *et al.*[17] showed that granular immune complex deposits composed of IgG and C3 in the TBM were detected in more than 80% of IgG4-TIN. C1q was also stained in a small number of cases. In a subclass analysis, Yamaguchi *et al.*[31] analysed five cases by immunofluorescence microscopy and found that all cases had IgG1 and IgG4 deposition in the TBM and interstitium. In addition, IgG3 deposits were found in three cases, C3 in three, and C1q in two.

## GLOMERULAR LESIONS

Although a variety of glomerular diseases have been reported to be associated with IgG4-RD, MGN is the most common (about 7%), having a specific significance [40–42,43^▪▪^,^44^–^46^]. MGN is classified into primary and secondary forms according to the presence or absence of an obvious cause. Anti-M-type phospholipase A2 receptor antibody, which is an important marker of primary MGN, is usually negative in IgG4-related MGN [43^▪▪^,^47^]. Granular deposits of IgG and C3 along the glomerular capillary walls, seen by immunofluorescence microscopy, are a typical feature. Interestingly, in the analyses of glomerular basement membrane (GBM) deposited IgG subclasses, IgG4 is the most dominant subclass in the great majority of patients and is usually accompanied by lower amounts of other IgG subclasses (Table 2) [40–42,43^▪▪^,^44^–^46^]. In contrast to primary MGN, granular C1q deposits are sometimes prominent, and some but not all cases have coexistent TBM deposits (Table 2). MGN is sometimes associated with IgG4-TIN, but some patients with MGN and IgG4-RD but without IgG4-TIN have also been reported [41,42,43^▪▪^,^44^]. MGN usually occurs simultaneously with IgG4-RD or becomes apparent during the course of already diagnosed IgG4-RD, but can precede IgG4-RD [45]. The response to corticosteroid therapy may differ between IgG4-related MGN and other organ lesions [46], and in some cases, proteinuria persists despite rapid disappearance of other IgG4-RD associated signs. As both MGN [48] and IgG4-RD [49,50] are thought to be associated with enhanced T helper type 2 (Th2) responses, a common pathogenetic role of Th2 responses is speculated.

Other glomerular lesions are classified into two subgroups according to the presence/absence of a predominance of Th2 responses. The association of Henoch–Schönlein purpura nephritis [51,52] or minimal change nephrotic syndrome [53], several cases of both of which have been published or presented, represent the former. The latter includes IgA nephropathy [35], membranoproliferative glomerulonephritis [54] and endocapillary proliferative glomerulonephritis [55].

## TREATMENT OF IgG4-RELATED KIDNEY DISEASE

A good and rapid response to corticosteroid therapy is a very important feature of IgG4-RD [1,2], and this has sometimes been used to confirm the diagnosis of type 1 AIP [56]. Steroid is the first-line therapy, and the administration of 0.6 mg/kg/day or 30 or 40 mg/day of prednisolone is recommended as the initial dose to induce remission in type 1 AIP [57,58]. The initial dose is continued for 2–4 weeks, and then tapered gradually (5 mg every 1–2 weeks) to a maintenance dose (5–10 mg/day). If the disease is refractory or frequently recurrent, addition of immunosuppressants such as azathioprine or rituximab is recommended [59,60^▪▪^].

Although in the vast majority of IgG4-TIN cases prompt recovery of renal function is achieved within 1 month of corticosteroid administration, in cases in which the eGFR has already decreased to less than 60 before treatment, only partial recovery of renal function is obtained (mostly in the first month and plateauing thereafter) [20^▪▪^]. The reasons for this can be explained through longitudinal pathological and imaging studies.

In histopathological examination, a re-biopsy study revealed that localized severe fibrosis became obvious in some parts after long-term corticosteroid maintenance therapy, although only minor abnormalities were seen in other parts [61].

**FIGURE 3:**
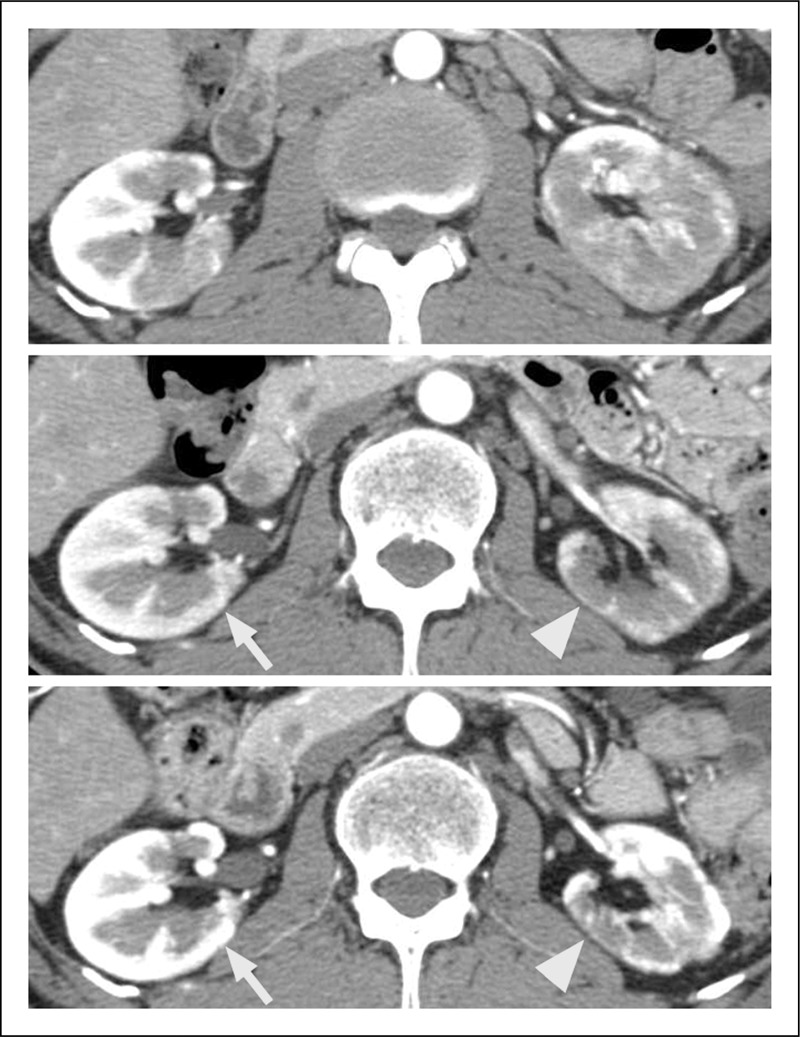
Longitudinal changes of imaging findings during corticosteroid therapy. Upper: Contrast-enhanced computed tomography (CT) scan before corticosteroid therapy shows multiple low-density lesions in the bilateral kidneys in a patient with IgG4-RKD. Middle: Two months after starting steroid therapy, contrast-enhanced CT scan shows complete recovery and disappearance of low-density lesions without atrophy in some areas of the kidney (arrow), while atrophic scarring starts to appear in other areas (arrowhead). Lower: Six years after therapy and still under steroid maintenance therapy, the area that showed early recovery maintains its normal appearance without atrophy (arrow), but the area where atrophic scarring started to appear shows progressive scarring with decreased enhancement (arrowhead) on contrast-enhanced CT scan.

In a longitudinal imaging study, contrast enhancement of the renal cortex recovered after therapy in almost all patients with multiple low-density lesions [20^▪▪^]. In particular, some areas of the kidney showed complete recovery and disappearance of low-density lesions without atrophy even with long-term administration of the maintenance dose of steroid (Fig. 3). In contrast, other areas of the kidney in the same patient developed atrophic scarring with decreased enhancement persisting (Fig. 3) [61]. This observation is very important because it implies that some areas of the kidney have reversible involvement and others irreversible involvement in the same patient, suggesting that the degree of fibrosis differs in individual parts of the kidney, and that a threshold of fibrosis exists, which when exceeded may push an area in the direction of irreversible fibrotic scarring. These findings might explain the reason for the early rapid but only partial recovery of renal function noted after steroid therapy.

## IS IgG4-RELATED DISEASE AN AUTOIMMUNE DISEASE?

There is controversy as to whether IgG4-RD is an autoimmune disease or not [1]. IgG4-related pancreatitis has been called AIP because Yoshida *et al.*[62], who proposed its concept, considered it to be an autoimmune disease on the grounds of hypergammaglobulinaemia, autoantibody seropositivity, frequent association with Sjögren's syndrome and primary biliary cirrhosis (PBC), and good responsiveness to corticosteroid therapy. However, more recently, greater experience has clarified that the diseases associated with it are not Sjögren's syndrome and PBC, but rather IgG4-related dacryoadenitis and sialadenitis and IgG4-related sclerosing cholangitis [63]. In contrast, the association of IgG4-RD with Sjögren's syndrome is very rare, despite Sjögren's syndrome being sometimes accompanied by other autoimmune diseases [64]. Next, many patients with IgG4-RD have been shown to have antinuclear antibodies (ANA) or rheumatoid factor. However, in more than half of patients with ANA, low titres (<x80) were present and most patients did not have disease-specific autoantibodies [47,63]. Therefore, some researchers have concluded that IgG4-RD might not be an autoimmune disease after all, but rather an allergic one. However, Mattoo *et al.*[65^▪▪^] recently showed that several single plasmablast-derived antibody clones established from patients with active IgG4-RD could react with autoantigens in the cytosole of Hep-2 cells. Therefore, further studies are needed to clarify whether disease-specific autoantibodies exist or not in IgG4-RD.

## IgG4-NEGATIVE IgG4-RELATED DISEASE

Recently, two cases of IgG4-negative IgG4-RD were reported [66^▪^,67^▪^]. These patients both showed typical clinical, imaging and histopathological features of IgG4-RD, despite the absence of any IgG4 involvement, that is with normal serum IgG4 levels and very few IgG4 and plasma cell infiltrates in the affected organs (Table 3). Interestingly, the favourable clinical course with a good response to corticosteroid seen in these patients resembles that in patients with IgG4-RD. Moreover, these two patients had biopsy-proven plasma cell rich TIN very similar to IgG4-TIN. Hart *et al.*[68] also showed three patients with type 1 AIP histologically but without serum or tissue IgG4 abnormalities. Thus, these cases suggest that a condition that closely mimics IgG4-RD may develop even in the absence of IgG4 and plasma cells.

## CONCLUSION

TIN with characteristic imaging findings is a typical manifestation of IgG4-RD in the kidney. MGN can be a manifestation of IgG4-RD, although a variety of glomerular diseases are known to accompany IgG4-TIN. Although IgG4 is a key molecule and abundantly present in both the serum and tissues in this disease, it is unknown whether IgG4 *per se* plays a crucial role in inducing multiple systemic lesions or is only a bystander. Analysis of many more cases, including ones with IgG4-negative IgG4-RD, with longer-term follow-up will be needed to define more precisely the role played by IgG4 in this disease.

## Acknowledgements

*We would like to thank Mr. John Gelblum for his critical reading of the manuscript*.

### Financial support and sponsorship

*This work was supported by Health and Labour Sciences Research Grants for the Study of Intractable Diseases from the Ministry of Health, Labour and Welfare, Japan*.

### Conflicts of interest

None.

## REFERENCES AND RECOMMENDED READING

Papers of particular interest, published within the annual period of review, have been highlighted as:

▪ of special interest
▪▪ of outstanding interest

## Figures and Tables

**Table 1:** Representative organ manifestations in IgG4-related disease

A. Organs adopted at the 1st international symposium in Boston in 2011	
Pancreas	Lymphoplasmacytic sclerosing pancreatitis
Eye/orbit/lacrimal glands	Dacryoadenitis/orbital inflammation/pseudotumour
Salivary glands	Sialadenitis/Mikulicz disease/Kuttner's tumour
Aorta/arteries	Aortitis/periaortitis/arteritis
Mediastinum/retroperitoneum/mesentery	Mediastinitis/retroperitoneal fibrosis/mesenteritis
Kidney	Tubulointerstitial nephritis/renal pyelitis
Pachymeninges/hypophysis/thyroid	Pachymeningitis/hypophysitis/Riedel thyroiditis
Lung	Lung disease/inflammatory pseudotumor
Pleura/pericardium	Pleuritis/pericarditis
Breast	Mastitis
Bile ducts/gall bladder/liver	Sclerosing cholangitis/cholecystitis/hepatopathy
Prostate	Prostatitis
Skin	Skin disease/pseudolymphoma
Lymph node	Lymphadenopathy
B. Organs newly recognized after the Boston meeting	
Nerve	Infraorbital nerve swelling
Paranasal sinus	Chronic rhinosinusitis
Testis/paratestis	Paratesticular pseudotumour
Ureter	Ureteritis
Urethra	Urethritis
Urinary bladder	Interstitial cystitis

**Table 2:** Immunoglobulin subclasses deposited on the glomerular basement membrane in IgG4-related membranous glomerulonephritis

	IgG1	IgG2	IgG3	IgG4	C3	C1q	TBM deposits	TIN with IgG4 and PC	References
83/M	2+	1+	–	2+	1+	–	IgG, IgG4, C3	Yes	Saeki *et al.* [40]
68/M	NA	NA	NA	+*	+*	NA	IgG, IgG4	No	Palmisano *et al.* [41]
54/M	±	–	3+	1+∼2+	±	3+	–	No	Cravedi *et al.* [42]
67/F	–	3+	–	1+	1+	-	C3	Yes	Alexander *et al.* [43^▪▪^]
67/M	±	±	–	3+	2+	–	–	Yes	Alexander *et al.* [43^▪▪^]
75/M	NA	NA	NA	+*	–	–	–	Yes	Alexander *et al.* [43^▪▪^]
53/M	±	1+	1+	3+	2+	–	–	No	Alexander *et al.* [43^▪▪^]
34/M	–	–	1+	2+	2+	±	± (focal)	No	Alexander *et al.* [43^▪▪^]
55/M	+*	NA	NA	+*	+*	NA	NA	No	Kanda *et al.* [44]
59/M	2+	1+	1+	2+	1+	–	–	(Only imaging)	Wada *et al.* [45]
69/M	NA	NA	NA	+*	–	+*	–	Yes	Miyata *et al.* [46]
80/M	NA	NA	NA	+*	+*	+*	NA	Yes	Miyata *et al.* [46]

IgG4 +PC, IgG4-positive plasma cells; NA, not available; TBM, tubular basement membrane; TIN, tubulointerstitial nephritis. ’+*’ indicates that intensity information is not available in the references.

**Table 3:** IgG4-negative IgG4-related kidney disease

Pt no.	Age/sex	Allergy	Histological findings	IgG4 IHC (cells/HPF)	Renal CT findings	IgG/IgG4 (mg/dl)	C3/C4 (mg/dl)	sCr (mg/dl)	Eo (/μl)	IgE(IU/ml)	Extrarenal lesions	Steroid response	References
1	56/M	none	pTIN, MGN	IgG4/IgG <2%	mLDL	4193/7.5	25/1	2.75	782	547	Sa, AIP, LN	Good	Makiishi *et al.* [66^▪^]
2	74/M	AR, BA, EP	pTIN	infrequent (<10)	mLDL	5593/20	40/1	0.71	1475	352	Sa, AIP, Lu, P, LN	Good	Hara *et al.* [67^▪^]

AIP, autoimmune pancreatitis; AR, allergic rhinitis; BA, bronchial asthma; Eo, eosinophil; EP, eosinophilic pneumonia; HPF, high power field; IHC, immunohistochemistry; LN, lymphadenitis; Lu, lung lesion; MGN, membranous glomerulonephritis; mLDL, multiple low-density lesions; P, prostatitis; pTIN, plasma cell rich tubulointerstitial nephritis; Sa, sialadenitis; sCr, serum creatinine.
